# University of Louisville

**Published:** 1855-08

**Authors:** 


### UNIVERSITY OF LOUISVILLE.

From the advertisement on the cover of this journal it will be seen that the lectures in the Medical Department of the University of Louisville commence on the last Monday in October, and that a preliminary course will be delivered during that month. The coming course will be the nineteenth in this institution. The school was organized in 1837 under the name of the *Medical Institute of Louisville*, and lectures were delivered during that winter to a class of eighty students. In 1845 the Medical Institute became a branch of the University of Louisville. It was liberally endowed by the city of Louisville. A noble edifice was erected, and a library and all the requisite apparatus purchased for its use. The matriculation fund has been devoted to the increase of its scientific furniture, and its museum, library, and chemical apparatus have been steadily increasing. Few schools anywhere have afforded students greater facilities for the study of anatomy. The Marine Hospital presents a fine field for the study of Clinical Medicine, and the Clinic at the University brings before students many interesting cases during the session.

In the preliminary course of lectures extending through the month of October, the Faculty have virtually lengthened the session to five months. It is believed that the lectures delivered in this preliminary course are equal in interest and value to any given during the winter, and students should therefore avail themselves of the advantages offered by this course.

The friends of the University will be gratified to learn that no changes have taken place in the Medical Faculty, in the last year, and that its organization promises now to be stable. The professors, with one exception, are all citizens of Louisville and not likely, soon, to seek to dissolve their connection with the school.

